# Non‐native rats detected on uninhabited southern Grenadine islands with seabird colonies

**DOI:** 10.1002/ece3.7313

**Published:** 2021-03-09

**Authors:** Wayne A. Smart, Natalia Collier, Virginie Rolland

**Affiliations:** ^1^ Department of Biological Sciences Arkansas State University State University Jonesboro AR USA; ^2^ Environmental Protection in the Caribbean Green Cove Springs FL USA

**Keywords:** Grenada, Introduced Species, Island, Rattus spp., Seabird

## Abstract

Seabirds are among the most endangered avian groups, with populations declining worldwide because of various threats, including invasive nest predators. Similar decreasing trends are occurring in the Southern Grenadines; however, the causes of decline remain uncertain, although non‐native rats have been suspected. Therefore, our objective was to determine whether non‐native rats are present on five Southern Grenadine islands that harbor seabird colonies, during May–July 2014–2017, using four methods (chew cards, tunnels, cameras, and questionnaires). Les Tantes East and Lee Rocks were the only two islands where cameras detected black rats (*Rattus rattus*). Although rat occupancy was low (0.125 ± 0.061) and the number of individuals and nesting attempts increased (except in 2017) for most species, the low detection probability and small number of nests prevented any inference about rat impact on seabirds. Rats might have affected seabird colonies, but other factors, such as seabird harvest, prey availability, or climatic fluctuations, could have also driven previous seabird population declines in the Southern Grenadines. However, non‐native rats are present and future research should focus on estimating their density and distribution on these and other islands of the region before an appropriate rat eradication program can be implemented.

## Graphical abstract

Causes of seabird decline remain uncertain in the Grenadines, although non‐native rats have been suspected. We surveyed five Southern Grenadine islands that harbor seabird colonies, during May–July 2014–2017, using four methods (chew blocks, tunnels, cameras, and questionnaires). Les Tantes East and Lee Rocks were the only two islands where cameras detected black rats (Rattus rattus) with an occupancy rate of 0.125.

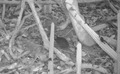

## 1 INTRODUCTION

Seabirds are long‐lived avian top predators that include many species at risk of extinction (Butchart et al., 2004; Velando et al., 2015), with 19% of the globally monitored seabird population in severe decline (Cuthbert & Sommer, 2003; McCauley et al., 2015; Paleczny et al., 2015). Seabirds spend most of their lives hunting on the open ocean, where they face various threats, including fisheries bycatch (Anderson et al., 2011; Barbraud et al., 2011), contamination from oil spills (e.g., Tran et al., 2014), heavy metal bioaccumulation through the marine food web (Burger & Gochfeld, 2000; García‐Tarrasón et al., 2013), and plastic pollution (Wilcox et al., 2015) leading to entanglement (Votier et al., 2011) or starvation (Pierce et al., 2004). Although seabirds spend most of their lives at sea, they return to land for breeding where they face additional anthropogenic threats. Historically, seabird harvest was probably the most important threat (Croxall et al., 2012), but legal protective efforts have reduced it in most regions (Devenish et al., 2009, but see Mondreti et al., 2018). Human presence in nesting colonies of uninhabited islands remains, however, a factor related to nest abandonment (Haynes‐Sutton et al., 2013) and human introductions of invasive mammals remain important drivers of population declines (Dias et al., 2019).

In general, invasive non‐native mammals represent one of the most serious threats to island birds (Bellard et al., 2016; Medina et al., 2011). Introduced herbivores, such as goats (*Capra* spp.) and rabbits (*Oryctolagus* spp.), can degrade nesting habitat by their foraging such that seabirds are unable to build nests in degraded areas (Glen et al., 2013). Non‐native vertebrate predators, such as rodents (*Rattus* spp. and *Mus musculus*) or cats (*Felis catus*), will depredate seabird nests and can decimate colonies, especially for ground‐ and burrow‐nesting species that have not evolved with such predators (Croxall et al., 2012; Fontaine et al., 2011; Jones et al., 2008; Wanless et al., 2007). Rats are particularly destructive for seabird colonies worldwide, including in tropical regions (Jones et al., 2008; Raine et al., 2020). In some cases, eradication can be an effective conservation tool; most seabird populations from which rats have been successfully eradicated respond positively (Bright et al., 2014; Brooke et al., 2017).

The Caribbean represents a region where the effects of introduced mammals on seabirds have been particularly detrimental (Hilton & Cuthbert, 2010; Schreiber & Lee, 2000). However, introduced mammalian predator threats remain unknown for most colonies in the West Indies (Schreiber & Lee, 2000). Schreiber and Lee (2000) recommended determining whether introduced predators were present on islands with seabird colonies because this knowledge could play a critical role in seabird conservation. For example, seabird declines have been documented in the Grenadines (Lowrie et al., 2012; Schreiber & Lee, 2000). Although the cause of this decline is unclear, Lowrie et al. (2012) suggested introduced rats as a potential cause of seabird nest failure in this region. Most of these islands are uninhabited, but fishers occasionally setting camp may have unintentionally brought rats onto some of the islands. Because the seabird breeding season coincides with the dry season, when food sources are more limited for terrestrial animals, introduced species such as rats may take advantage of seabird eggs and chicks as alternative food sources (Caut et al., 2008).

Thus far, evidence of rats on these islands is limited. Collier (2014) did not find conclusive evidence of rat presence on some of the offshore islands of St. Vincent and the Grenadines. However, Collier (2014) only surveyed a small proportion of islands (4% of 90 islands) and the surveys were only conducted for two days per island, which could have limited the chances of detecting rats, despite the 33–62 baited chew cards, 1–3 tracking tunnels, and 0–1 camera deployed on each island. Thus, to ascertain that rats are not involved in the decline of seabirds in the Grenadines, further, more intensive efforts are necessary. In this study, our goal was to determine whether non‐native rats are present on uninhabited Grenada islands that host seabird colonies, and if present, we wanted to obtain an occupancy estimate. We focused on five accessible islands, two of which are of regional (Diamond Rock) or global (Les Tantes East) importance for seabird colonies; the other three could be of importance but were not (or partially) included in the 2010 survey (Lowrie et al., 2012). Overall, Collier (2014) did not assess any of the five selected islands for rat presence. Additionally, although the 2010 survey included 5–7 wooden sprung baited traps near colonies on the two islands of importance, these were only deployed for 4.75–7 hr during the day when rats are less or not active (Lowrie et al., 2012). This research provides information that should assist (a) conservationists in making plans to further seabird conservation in the Grenadines specifically, and in the Caribbean in general, and (b) working groups in assessing threats and population trends of seabird species globally.

## 2 METHODS

### 2.1 Study area

The Grenadines consist of about 90 relatively small oceanic offshore islands formed between the mainlands of St. Vincent [13°15′11N, 61°11′00W] and Grenada [12°07′05N, 61°40′41W] from the convergence of the subduction of the North American tectonic plate under the Caribbean plate. In total, these islands cover 130 km^2^ of land surface. Shorelines are generally irregular, blocked by shallow shelves and coral reefs. The Grenadines are typically exposed to northeast prevailing winds and temperatures average 24°C year‐round. Islands experience seasonal drought and rainfall with average monthly precipitation of 6.2 cm during the dry season and 23.5 cm during the rainy season. Vegetation cover is dominated by deciduous plants with open canopies generally not exceeding 6 m, including Indigoberry (*Randia aculeata*) and succulent herbaceous shrubs such as Sea Grape (*Coccoloba uvifera*), which grow on rocky substrate. Other common plants include *Acacia* spp. and cacti, such as *Pilosocereus royeni*. Most of the island soil was rocky except for some sandy shores and areas of soft, dark‐colored soil derived from volcanic material. Humans do not reside on any of the study islands, but fishers and recreationists sometimes set temporary camps.

**FIGURE 1:**
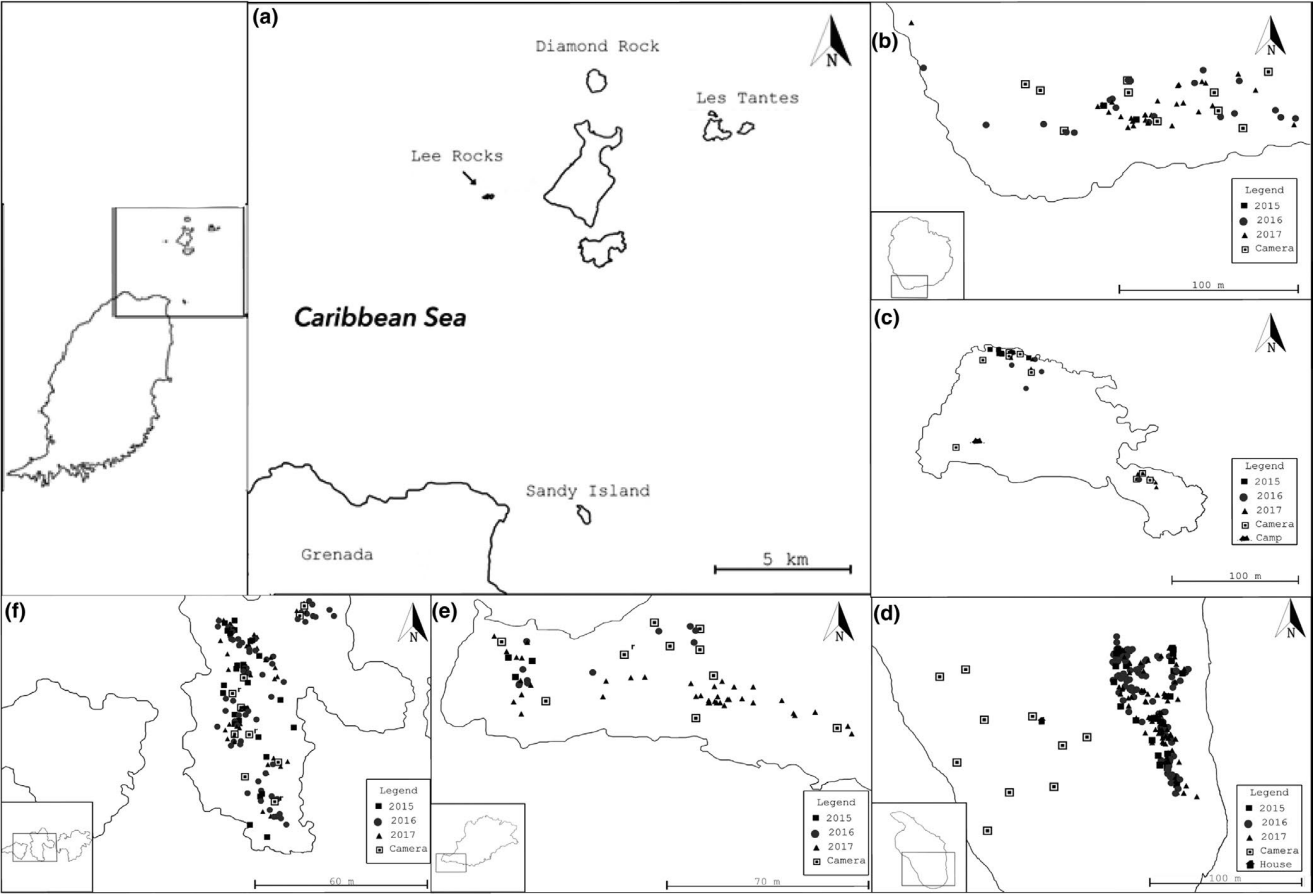
Grenada and South Grenadine islands [12°16′22.33″N, 61°37′17.63″W] (a) and locations of seabird nests and 2017 cameras on the study islands of Diamond Rock (b), Les Tantes North (c), Sandy (d), Les Tantes East (e), and Lee Rocks (f). “*r*” indicates cameras that detected rats for Les Tantes East and Lee Rocks

We conducted the study from early May to late July of 2014–2017 at five Grenadine islands (Figure 1): Diamond Rock, Les Tantes North (also known as Grass Island), Les Tantes East (also known as Petite Tante), Lee Rocks, and Sandy Island. Sandy island was only included as a study site starting in 2015. These 0.4–22.1 ha islands sit 1.8–11.8 km north of mainland Grenada (Table 1). In 2009–10, Lowrie et al. (2012) surveyed Diamond Rock and Les Tantes for which they listed six breeding species (Table 2): Bridled Tern (*Onychoprion anaethetus*), Brown Noddy (*Anous stolidus*), Laughing Gull (*Leucophaeus atricilla*), Red‐billed Tropicbird (*Phaethon aethereus*), Brown Booby (*Sula leucogaster*), and Red‐footed Booby (*Sula sula*). For the latter three species, Diamond Rock and Les Tantes are of regional and global importance, respectively (Lowrie et al., 2012). In addition, The Sisters, near Lee Rocks, have potential to harbor a colony of Sooty Terns (*Onychoprion fuscatus*). Although the International Union for Conservation of Nature Red List (BirdLife International, 2018a, 2018b, 2018c, 2018d, 2018e, 2019a,2019b) lists all species’ status as least concern, the tropicbird and the two booby species are decreasing partly because of introduced species, including rats (BirdLife International, 2018d, 2018e, 2019b). Rats also impact colonies of Sooty Terns and Brown Noddies (BirdLife International, 2018a,2018b).

**TABLE 1:** Grenadine islets characteristics and detection of non‐native rats in 2014–17. Les Tantes are composed of three islets, including East and North. The shortest distance is from each islet to the coast of mainland Grenada

Island	Area (ha)	Shortest distance to Grenada (km)	Rat presence (X) by method of detection			
			Chew card	PVC tunnel	Camera	Fishers survey
Diamond Rock	22.1	11.2				
Lee Rock	0.4	7.7	X		X	
Les Tantes	1.7 (East) 7.5 (North)	11.8			X	X
Sandy	8.6	1.8				

**TABLE 2:** Nesting colonies of five seabird species on five Grenada Grenadine islands. No. pairs represent the number of breeding pairs surveyed in January‐March 2009–10 (Lowrie et al., 2012), whereas No. indiv and No. nests provide minimum and maximum for the number of individuals (counted from a boat circling the island in August) and nests (monitored from May‐July) in chronological order for 2014–2017

		BRTE	BRBO	BRNO	LAGU	RBTR	RFBO	MAGA
Diamond	No. pairs	P	388	2	1,200	148	656	NP
	No. indiv	32–13–1–0	60–52–99–**291**	26–52–**123–**60	14–12–44–84	0–14–0–0	0–0–0–115	0–0–**16**–14
	No. nests	0–0–0–0	3–1–11–**17**	2–0–0–1	3–0–0–1	0–**2**–0–0	‐	‐
Les Tantes	No. pairs	13	252	12	44	77	2,101	NP
East	No. indiv	21–12–2–5	0–0–0–76	32–13–47–4	6–5–23–93	**15**–0–4–9	352–**590**–193–360	0–0–0–15
	No. nests	5–2–4–**10**	0–0–1–7	4–0–0–0	0–0–0–19	1–1–1–1	‐	‐
North	No. nests	7–3–5–5	0–0–0–1	3–**5**–0–0	0–0–0–6	0–0–**2**–1	‐	‐
Lee Rocks	No. indiv	6–26–**36**–13	2–26–67–19	10–33–99–74	84–18–52–71	0–2–0–0	0–0–0–1	0–0–0–0
	No. nests	1–0–2–3	1–1–4–2	1–1–2–2	13–30–51–86	0–0–**2**–1	‐	‐
Sandy	No. indiv	30–0–0–12	0–0–0–1	0–0–0–0	102–18–89–**302**	0–0–0–0	0–0–0–0	0–0–0–3
	No. nests^$^	0–0–2–0	0–0–0–0	0–0–0–0	33–**102**–90	0–0–0–0	‐	‐

Note

Bold numbers highlight the island where the highest counts (individuals or nests) were recorded for a given species. Species were Bridled Terns (BRTE), Brown Boobies (BRBO), Brown Noddies (BRNO), Laughing Gulls (LAGU), and Red‐billed Tropbicbirds (RBTR). Nests were not accessible for Magnificent Frigatebirds (MAGA) or Red‐footed Boobies (RFBO); RFBO individuals were counted from an adjacent island for ten minutes. Les Tantes combined Les Tantes East and Les Tantes North. P and NP stand for present and not present, respectively.

Two non‐native rat species could potentially be found on our study islands: black (*Rattus rattus*) and brown (*R. norvegicus*) rats. Brown rats are present on mainland Grenada (e.g., Coomansingh‐Springer et al., 2019) and black rats were reported on the offshore island of Cariacou (Pendleburry, 1974). There is no record of native predators of seabirds nesting on the five uninhabited study islands. Potential native predators of adult seabirds, including Broad‐winged Hawks (*Buteo platypterus*), occur on mainland Grenada, but their presence has not been reported on any of the study islands, except for one sighting of a Peregrine Falcon (*Falco peregrinus*) on Diamond Rock (Collier, personal observation). On larger neighboring islands (e.g., Ronde Island), the common opossum (*Didelphis marsupialis*) is a known nest predator. Snakes, including the Barbour's tropical racers (*M. bruesi*) and tree boas (*C. hortulanus*), which may prey on both nestlings and eggs (Malhotra & Thorpe, 1999), inhabit both Grenada and several Grenadine islands, but remain unidentified for our study sites.

### 2.2 Breeding colony size estimation

Starting on 16 or 17 May of each year, we searched for active nests (i.e., with one or more eggs/chicks) in accessible areas of all five study islands. We then monitored them weekly and searched for new nests until the end of July. The number of nesting attempts was used as a proxy for the number of breeding pairs. For some species, monitoring procedures required reaching under the parent to search for eggs. Specifically, Red‐billed Tropicbird nests were approached from under the nest or at nest level to make the tending adult fully aware of our presence and to minimize stress (Del Nevo, 2010).

In addition to the number of nesting attempts, each year, during the first week of August (when most species are finished nesting), we conducted an in‐transit count of all birds (incubating, loafing, or flying over the colony) visible from a boat with binoculars by circling each island once, ~30 m offshore, at 6–10 knots (11–18.5 km/h). We chose in‐transit counts over other available methods because they are more feasible for citizen scientists to easily replicate and to compare future counts to ours, should a long‐term monitoring program be implemented in the Grenada Grenadines. This in‐transit count method is already used in a citizen‐based monitoring program in the St Vincent and the Grenadines (Mackin et al., 2016).

### 2.3 Predator presence assessment

We determined the presence of potential predators on each island using chew cards (Oberg et al., 2014), tracking tunnels (Blackwell et al., 2002), and cameras (Rendall et al., 2014). However, we did not use all methods every year. We did not obtain cameras until 2015 and we stopped using PVC tunnels and chew cards in 2016–17 because baits were consumed by ants or became waterlogged with rain and because we could not ascertain rat species identity from bite marks (Sweetapple & Nugent, 2020).

We deployed seven indicator chew cards (i.e., squares of baited corrugated plastic), at least 5 m apart, haphazardly throughout each island to sample shores, colonies, and other inland areas. We monitored them weekly from mid‐May to the end of July. To avoid attracting crabs (Oberg et al., 2014), we elevated all baited indicators at about 30 cm with flagged metal stakes through the center. We also deployed three PVC tracking tunnels biweekly, at one island at a time. These tracking tunnels were baited and placed on top of 4‐L buckets. We used the same bait (a mix of flour, coconut, and peanut butter) for both chew cards and tracking tunnels.

Second, we attached 10 motion‐activated infrared cameras (five Bushnell 14MP Trophy cam HD Aggressor No Glow, Overland Park, MO; three Bushnell 12MP Trophy cam HD, Overland Park, MO; and two Cuddeback 20MP long‐range IR, De Pere, WI) to trees or their stumps within or nearest to colonies (Figure 1), 0.5–3 m off the ground and 3 m from a 12‐cm‐long PVC pipe stuffed with bait and tied to a tree. Because Sandy Island lacked trees close to the colony, we placed the cameras farther west around an abandoned house where rats could find shelter (Figure 1d). All cameras had a trigger speed of 0.2–0.3 s and we set them to capture three photos every 15 s, once triggered, with a night‐vision shutter on high to minimize blurriness. The default auto setting was kept for the sensor level.

In 2017, we used all cameras on each island for two consecutive weeks. However, in 2015, we only had one camera, deployed 24–29 May on Sandy and 23–30 June on Lee Rocks. In 2016, we had acquired four cameras and deployed one on each island from 24 May to 21 July. Additionally, on all visits to the islands, we also opportunistically looked for tracks and scats on using the same route every year.

Finally, we designed a survey that we made available from 15 June to 30 July (2015–2017), a period outside the peak of the fishing season (FAO, 2018) so fishers could give us a taxi ride to the study islands. Copies of the survey were left at the Fisheries Division Office of Sauteurs, Grenada, near the closest seaport to the study islands. Most fishers and recreationists who use the study islands would enter and leave mainland Grenada through this seaport. The survey included the following five questions about their knowledge and observations of predators on the islands:

1. List all the predators of seabirds that you recall encountering while in the Grenadines
2. Have you encountered rats on any of the seabird sites?
3. Which Grenadine islands have you encountered rats?
4. When last did you encounter rats during your visit to the Grenadines?
5. What level of impact do you think rats have on nesting seabird colonies?

The Arkansas State University Institutional Animal Care and Use Committee [750611–1] and Institutional Review Board [750611–2] have approved these field and survey protocols. All human subjects have signed an informed consent form before taking the survey and the research was conducted in accordance with the Helsinki Declaration of 1975, revised in 2008.

### 2.4 Data analyses

We used program PRESENCE (https://www.mbr‐pwrc.usgs.gov/software/presence.html) to estimate the probability of non‐native rats being present (also called occupancy). This software was developed to estimate the probability that a site is occupied by a species of study, after correction for imperfect detection probability (MacKenzie et al., 2002). Because this method relies on repeated surveys at multiple sites and we deployed only one camera (i.e., surveyed only one site) per island in 2015 and 2016, we analyzed only camera data from 2017. We marked each week of the 2017 nesting season as “1” if a rat was detected by a camera, “0” otherwise. We generated estimates of occupancy (ψ) and detection (*p*) using single‐season models, accounting for false absences (USGS, 2017). In a two‐step approach, we first compared models with survey‐specific (i.e., detection probabilities differ for each week) and constant *p* (i.e., same detection probability for all weeks) while keeping ψ constant. Then, starting from the best general *p* structure, we compared models with constant and site‐specific ψ.

## 3 RESULTS

### 3.1 Breeding colony size

We located 241.5 ± 48.3 nests annually for a total of 566 nesting attempts over the 4‐year study period (Figure 1) with most attempts in 2017 (*n* = 253) and fewest in 2014 (*n* = 43). The number of individuals and nesting attempts increased for Brown Boobies and Laughing Gulls, whereas these numbers remained relatively stable for Red‐billed Tropicbirds (Table 2). Although the number of nesting attempts was similarly stable for Bridled Terns, the number of individuals during in‐transit counts declined from 2014 to 2017 (Table 2). For Brown Noddies, the number of nesting attempts tended to decline, and the number of individuals dropped in 2017 (Table 2). Laughing Gulls ranked as most productive species (with an annual 108.5 ± 42.2 attempted nests), followed by Brown Boobies (13 ± 6 nests) and Bridled Terns (12.3 ± 2.7 nests); Brown Noddies (5 ± 1.6 nests) and Red‐billed Tropicbirds (3.0 ± 0.8 nests) were the least productive.

Diamond Rock harbored the most seabirds; in‐transit counts included all species, although we found no Bridled Tern nests and Red‐billed Tropicbirds nested there only in 2015 (Table 2). Notably, of all five study islands, Diamond Rock consistently had the highest counts of Brown Booby nests and individuals, but also had a record number of Brown Noddies in 2016. Every year except 2015, we counted the largest number of Bridled Terns at Lee Rocks but never found more than 3 nests. All five monitored species nested at Lee Rocks and we recorded the second highest numbers of flying Brown Boobies and Brown Noddies and the second highest number of Laughing Gull nests (Table 2). Sandy Island was the most important for nesting Laughing Gulls but did not seem important for any of the other species despite two Bridled Tern nests and rare sightings of Brown Boobies and Magnificent Frigatebirds (*Fregata magnificens*) in 2017 (Table 2). We also observed a few Magnificent Frigatebirds at Diamond Rock and Les Tantes East, but only in the last 2 years. In‐transit counts suggested that Les Tantes (particularly Les Tantes East) was most important for Red‐Footed Boobies, although they also attracted Laughing Gulls, Brown Noddies, Bridled Terns, and Red‐billed Tropbicbirds. In fact, Les Tantes held the highest number of Bridled Tern nests and the highest number of flying Red‐billed Tropbicbirds (Table 2). We did not observe any Sooty Tern.

### 3.2 Rat presence

**FIGURE 2:**
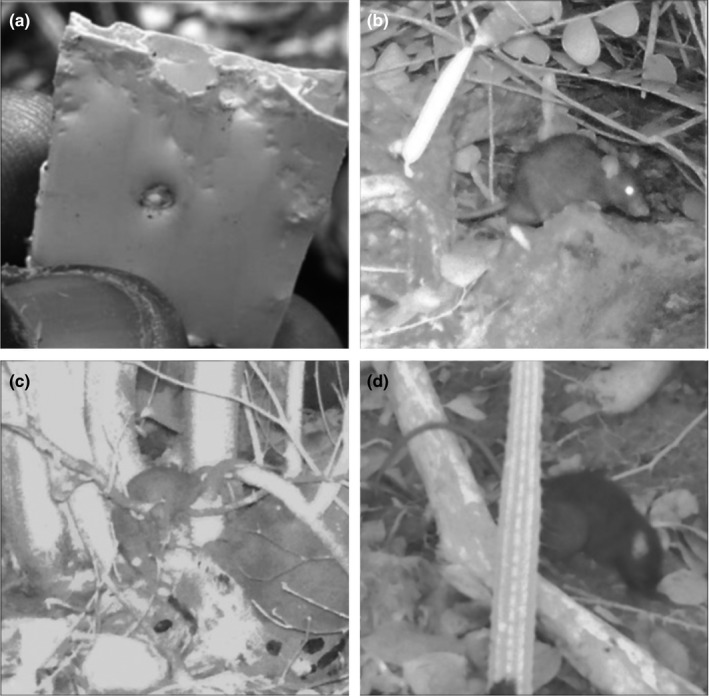
Evidence of rat presence on Grenadine islands: Bite marks on corrugated plastic chew card on Lee Rocks in 2015 (a); Individuals of *Rattus rattus* caught on camera on Lee Rocks in 2016 (b) and 2017 (c), and on Les Tantes East in 2017 (d)

Rat detection methods were unequally successful (Table 1). We never identified any rodent track or scat. Chew cards and tracking tunnels were unsuccessful in 2014. In 2015, one chew card on Lee Rocks showed ragged and incisor‐pair marks, indicative of rat presence (Figure 2a), although we could not identify the marks to species. Similarly, cameras recorded the presence of rats on Lee Rocks in 2016, and again on both Lee Rocks and Les Tantes East in 2017 (Figures 1 and 2). From one photo from Lee Rocks in 2016, the rat was identified as *R. rattus* based on relative tail length and ear size (Swinnerton, pers. comm.), but we were unable to identify all individuals to species because of rat position or photo quality. The detection probability was constant (0.125 ± 0.061; Table 3), but the best occupancy model (i.e., ψ (site), *p* (.); Table 3) indicated a difference among sites. Rat occupancy was higher on Lee Rocks (0.544 ± 0.25) than Les Tantes East (0.123 ± 0.12) but was not estimable for the other study islands.

**TABLE 3:** Model selection of rat occupancy (ψ) and detection (*p*) for all five study sites in the southern Grenadines in 2017. Occupancy was modeled as constant (.) or as a function of site, whereas *p* was modeled as constant or as a function of survey. AIC, ΔAIC, AICwt, No. Par stands for Akaike information criterion, difference in AIC between a given model and the model with the lowest AIC, AIC weight, and number of parameters, respectively

Model	AIC	ΔAIC	AICwt	No. Par
ψ (site), *p* (.)	41.760	0.000	0.431	6
ψ (.), *p* (survey)	46.410	4.650	0.042	11
ψ (.), *p* (.)	47.180	5.420	0.028	2

**FIGURE 3:**
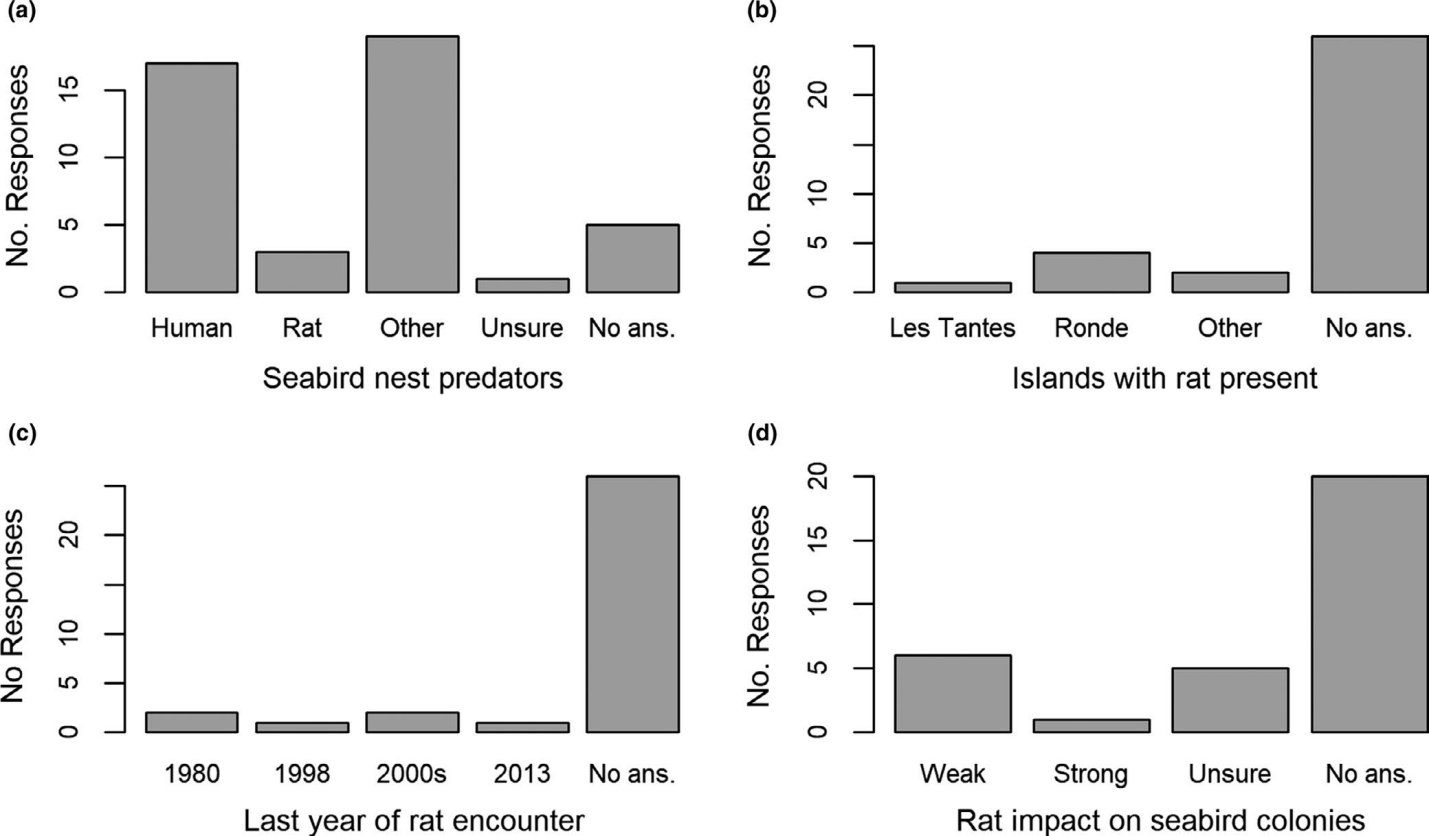
Tallied responses of 32 surveys to four survey questions to Grenadians fishers and recreationists from 2015 to 2017. Questions were: (a) List all the predators of seabirds that you recall encountering while in the Grenadines, (b) Which Grenadine islands have you encountered rats?, (c) When last did you encounter rats during your visit to the Grenadines?, and (d) What level of impact do you think rats have on nesting seabird colonies? The category “Other” in question a represents other predators (i.e., snakes [*n* = 4 respondents] and opossum [*n* = 5]) and nonpredators of seabirds (i.e., goats [*n* = 5] and iguanas [*n* = 13])

We received 32 responses from the self‐administered surveys across the 4‐year study period. When respondents provided multiple answers to a question (e.g., more than one type of predator to the first question), we tallied all their answers, which resulted in more than 32 answers. Eighteen percent of respondents (*n* = 6) have encountered rats in the southern Grenadines (Figure 3a), including at one of our study sites (i.e., Les Tantes; Figure 3b). Their last encounters dated back to 1980 and as recently as 2013 (Figure 3c). However, only three respondents reported rats as a predator of seabird nests, whereas 17 respondents listed humans (Figure 3a). Additionally, although most (78%) respondents either did not provide an answer (*n* = 20) or were unsure (*n* = 5) about the impact of rats on nesting seabird colonies, six (19%) believed it to be weak while only one considered that rats have a strong impact on nesting seabird colonies (Figure 3d).

## 4 DISCUSSION

Seabird populations are declining globally (Croxall et al., 2012), including in the Caribbean region (Lowrie et al., 2012) and knowledge of nest threats is important to make appropriate conservation and management decisions for local seabird populations. Our study confirmed, on at least two of the five study islands, the presence of non‐native rats that could represent a threat to seabird colonies nesting in the Southern Grenadines. These data are valuable for informing future seabird conservation on these understudied islands.

As noted by Lowrie et al. (2012), Les Tantes East is an important island for breeding seabirds, particularly for Red‐footed Boobies, but our survey confirms that five other species nest there, including Red‐billed Tropicbirds. Lee Rocks, which had not been surveyed before attracted the same five species for nesting, despite its smallest size. We also confirmed nesting Bridled Terns on Diamond Rock which had been previously noted as present but for which no count had been reported. We did not detect rats on Diamond Rock, Sandy, or Les Tantes North. However, absence of detection does not equate absence of rats; the low capture probability estimated by our occupancy model suggests that the number of cameras may not have been enough for detection and we cannot infer rat absence (Pellet & Schmidt, 2005).

We detected rats on two islands, Lee Rocks and Les Tantes East. Perhaps not coincidentally, Les Tantes East was also the only study site where survey respondents encountered rats. Although we could confirm their presence on these two islands, their impacts on nesting seabirds are unclear. First, most respondents did not believe these rats to have a strong impact on seabird colonies, but their observations and understanding of ecological impacts of rats may be limited. Second, our counts at Les Tantes East suggest a decline for the two booby species and for Red‐billed Tropbicbirds from the 2009–10 survey (Lowrie et al., 2012; Table 2), but the numbers are not directly comparable because the surveys were not conducted at the same time of year or with the same method. Third, across our 4‐year study period, the number of nests on Lee Rocks and Les Tantes East remained stable or increased for all species except Brown Noddies on Les Tantes East. Rats may be preying on other available wildlife, such as hermit crabs (Pitman et al., 2006) or there may have been too few nests of each species to detect an impact of rats. The only clear increase was for Laughing Gulls on Lee Rocks, but this species is not as vulnerable to rats as smaller, hole‐nesting Laridae species (e.g., Bridled Terns) or Red‐billed Tropicbirds (Jones et al., 2008). However, even the bigger, less accessible, tree‐nesting Red‐footed Booby remains at risk of nest depredation by rats because both *Rattus* species can climb trees to acquire food (Foster et al., 2011). Perez‐Correa et al. (2020) also suggest that abundance of Red‐footed Booby and Brown Noddy at sea is related to rat presence on nearby islands.

Although one rat was identified as *R. rattus* and this species has been involved in at least half of island invasions by rats (Russell et al., 2008), we cannot exclude the presence of *R. norvegicus*. *Rattus norvegicus* can swim 1,000 m comfortably in temperate water and up to 2,000 m under suitable conditions; *R. rattus*, although a weaker and less inclined swimmer, can travel up to 1,000 m by swimming or drifting on debris in open water (Russell et al., 2008). Therefore, rats—whether they were *R. rattus* only or a mix of both—could have swum to Lee Rocks from Ronde Island where survey respondents reported having seen rats. Ronde Island is ~700 m shore‐to‐shore from The Sisters, itself ~700 m from Lee Rocks. Additionally, *R. norvegicus* was associated with an occupancy rate of 0.4–0.6 on islands that are ~1,000 m from the main rat population source (Tabak et al., 2015). This estimate is consistent with our rat occupancy estimate for Lee Rocks. However, we did not detect rats on Diamond Rocks, which is ~800 m from the closest shore of Ronde Island, whereas Les Tantes where we detected rats (albeit at a smaller rate) is 1.4 km from Ronde Island. An equal or more likely explanation is that rats came from boats of fishers who sometimes set temporary camps on the islands.

In addition to tracking tunnels, chew cards, and cameras, we opportunistically looked for tracks and scats. Although tracks are not detectable on rocks or sand, we did not find any on dark soil, nor did we find any scat, suggesting that the rat population size may be low. Alternatively, the probability of detecting an individual on camera may have been lower on Les Tantes East because Les Tantes East was larger than Lee Rocks while the number of cameras remained the same on both islands. This sort of dilution effect could also explain why we did not detect rats on other islands, particularly Sandy Island, the largest of our study sites, despite having been historically inhabited. Although two weeks of sampling for each island is likely enough to obtain accurate estimates of rat occupancy (Christie et al., 2015), the limited number of cameras may explain the low detection probability. Nonetheless, this detection technique was still more successful at detecting rats than chew cards or tracking tunnels. Overall, these corrugated plastic pieces attracted more ants. Other studies also reported nontarget animals, such as ants (Collier, 2014) and land crabs (Oberg et al., 2014). Only one chew card presented evidence of rat presence on Lee Rocks. Perhaps, elevating chew cards to 30 cm off the ground excluded not only hermit crabs but also rats, reducing their effectiveness for rat detection. Finally, tracking tunnels, regardless of their number and placement, do not perform as successfully when rodent populations occur at low density (Blackwell et al., 2002), which might have been the case at the five study islands.

Now that the presence of rats—including *R. rattus*, the invasive rat species with the highest impact on seabird (Jones et al., 2008)—is confirmed on the islands, not only eradication becomes an important management consideration, but sampling effort should also be extended to other islands that have not been surveyed to improve our understanding of rat impact on seabird colonies in the Caribbean and to inform threat assessments for seabird status worldwide (Schreiber & Lee, 2000). On each island, the sampling effort should be increased to cover the whole area by increasing the number of cameras to determine rat distribution and abundance, two necessary parameters to increase the effectiveness and unintended consequences of any eradication program (Sweetapple & Nugent, 2011). Timing would also need to be considered to minimize nontarget mortality (Black et al., 2017). Finally, because poisons can persist in the environment, monitoring should be conducted post‐treatment to not only validate its effectiveness on non‐native rats, but also the effects on native species population growth (Brooke et al., 2017; Martin & Richardson, 2019). Alternatively, rapid eradication assessment is a tool that can estimate the probability of success of the eradication program to confirm rat absence (Kim et al., 2020; https://rea.docker.stat.auckland.ac.nz/).

Although non‐native rats may have caused some nest failures, other factors, such as prey availability, climate change, or seabird harvest (Dias et al., 2019), could have contributed to past seabird population declines at the study sites. Besides non‐native rats, seabird harvest had also been suspected as a potential cause of decline of seabird colonies in the Grenadines (Devenish et al., 2009; SUSGREN, 2013). The survey results would support this hypothesis as half the respondents on the survey listed humans as a predator along with rats. Additionally, Smart et al. (2020) reported that seabird harvest still occurs in the southern Grenadines, albeit less today possibly because of higher fuel prices. Therefore, future research should also focus on monitoring seabird colonies on the study and other understudied islands of the Caribbean, estimating cause‐specific nest failures, and assessing the extent and potential impact of rat presence and seabird harvest on seabird nesting productivity (Schreiber & Lee, 2000).

## CONFLICT OF INTEREST

Authors have no conflict of interest to declare.

## AUTHOR CONTRIBUTION

**Wayne A Smart: Formal analysis (equal); Funding acquisition (equal); Investigation (lead); Methodology (equal); Writing‐original draft (lead); Writing‐review & editing (supporting). Natalia Collier: Conceptualization (equal); Funding acquisition (equal); Resources (supporting); Writing‐review & editing (equal). Virginie Rolland: Conceptualization (equal); Formal analysis (equal); Funding acquisition (equal); Methodology (equal); Supervision (lead); Writing‐review & editing (equal).**

## Data Availability

Original responses from human participants cannot be provided to ensure confidentiality and follow the protocol approved by the Arkansas State University Institutional Review Board. However, compiled answers from these questionnaires as well as camera rat survey data and photos, seabird nest data, and seabird in‐transit count data used in this manuscript are available in the Dryad Digital Repository, at https://doi.org/10.5061/dryad.547d7wm7j.
